# Adverse effects of type 2 diabetes mellitus on ovarian reserve and pregnancy outcomes during the assisted reproductive technology process

**DOI:** 10.3389/fendo.2023.1274327

**Published:** 2023-11-15

**Authors:** Xue Qin, Junhong Du, Ruifen He, Yi Li, Qinying Zhu, Yaxi Li, Hongli Li, Xiaolei Liang

**Affiliations:** ^1^ The First Clinical Medical College of Lanzhou University, Lanzhou, Gansu, China; ^2^ Department of Obstetrics and Gynecology, The First Hospital of Lanzhou University, Lanzhou, Gansu, China

**Keywords:** retrospective cohort study, type 2 diabetes mellitus, ovarian reserve, anti-mullerian hormone, embryonic development, assisted reproductive outcomes

## Abstract

**Objective:**

To study the effect of type 2 diabetes mellitus(T2DM)on overall ovarian reserve and pregnancy outcomes during assisted reproductive technology (ART) among childbearing infertile women.

**Design:**

Retrospective cohort study.

**Setting:**

The Reproductive Medicine Special Hospital, The First Hospital of Lanzhou University, between January 2019 and December 2022.

**Patient(s):**

A total of 265 infertile female patients aged 20–45 years who underwent *in vitro* fertilization-embryo transfer (IVF-ET), intracytoplasmic sperm injection-embryo transfer (ICSI-ET), or rescue intracytoplasmic sperm injection-embryo transfer (RICSI-ET) in the first fresh cycle.

**Intervention(s):**

None.

**Main Outcome Measure(s):**

Serum Anti-Müllerian Hormone (AMH) levels, clinical pregnancy rate (CPR), live birth rate (LBR), and abortion rate (AR) in the T2DM group and non-T2DM group.

**Result(s):**

Patients with T2DM showed statistically decreased levels of AMH compared to the non-T2DM group. During ovarian stimulation, those with T2DM required significantly higher total and initial doses of gonadotropin (GN), although they had fewer retrieved oocytes and worse pregnancy outcomes than the non-T2DM group. Multivariate logistic regression analysis adjusting for confounding factors showed that T2DM alone was an independent risk factor for CPR and LBR (adjusted odds ratio [a OR], 0.458, adjusted 95% confidence interval [CI], 0.235-0.891, P = 0.022; a OR, 0.227, 95% CI, 0.101-0.513, P<0.001; respectively), and the abortion rate in the T2DM group was 3.316 times higher than the non-T2DM group(a OR, 3.316, 95%CI, 1.248-8.811, P = 0.016);

**Conclusion:**

Infertile patients with T2DM have decreased ovarian reserve, and T2DM has a deleterious impact on clinical pregnancy outcomes during the ART process compared with non-T2DM infertile women.

**Capsule:**

Infertile women with T2DM have decreased ovarian reserve and pregnancy outcomes during the assisted reproductive technology process compared with non-T2DM infertile women.

## 1 Introduction

Type 2 diabetes mellitus (T2DM) accounts for 90–95% of all diagnosed cases of diabetes, and, in parallel with the worldwide escalation in the prevalence of obesity, T2DM is becoming increasingly common on a global scale (1). As a common endocrine disease, T2DM is characterized by insulin resistance, relatively insufficient insulin secretion (2), and elevated fasting and postprandial blood glucose (3). The main complications of T2DM include cardiovascular disease (4), diabetic retinopathy (5), and neuropathy (6), as well as dysfunctional immunity (7) and a chronic inflammatory state (8). It is widely recognized that T2DM and its complications will bring profound psychological and physical distress to patients and put a huge burden on society (4).

In addition, studies have demonstrated that reproductive dysfunction is also a common but little studied complication of diabetes (9). Due to the state of hyperglycemia, women with diabetes may have substantially decreased fecundability (10) and higher proportions of spontaneous losses (11). Type 1 diabetes mellitus (T1DM), which is characterized by β-cell destruction and an absolute deficiency of insulin secretion (12), may lead to the accumulation of advanced glycation receptors and products (13) and appear more likely to have reproductive disorders (14), manifested by polycystic ovary syndrome (PCOS) (15), irregular menses (16, 17), and subfertility (18, 19). Studies also reported that the rates of spontaneous abortion among women with T1DM are increasing (20). T2DM, also known as non–insulin-dependent diabetes (21), although it has a shorter duration of diabetes than T1DM, both are equally at risk for reproductive disorders (9). There is growing evidence that T2DM impairs the function of the female reproductive system and negatively affects fertility (22). A recent review reported that T2DM was associated with lower rates of fertility (6). Another large prospective cohort study also found that women with T2DM prior to childbearing had a higher risk of miscarriage and infertility (23).

As the prevalence of T2DM rises sharply globally, especially among young people, more women of reproductive age will encounter severe challenges to ovarian reserve and pregnancy outcomes (6). This means that the increasing childbearing women with T2DM need to rely on assisted reproductive technology (ART) to complete fertility. However, comprehensive and holistic assessments of pregnancy outcomes in women with T2DM during the ART process have been rarely conducted (24), and few studies have illustrated associations between T2DM and reproductive outcomes in terms of pregnancy, live birth, or miscarriage, which attracted our attention.

Anti-Müllerian Hormone (AMH), a glycoprotein produced exclusively by granulosa cells of ovary (25, 26), is the most clinically useful marker of ovarian reserve (27) because of its strong correlation with primordial follicle pool and aging of ovary (28). An experimental study in a rat model revealed that AMH levels in the healthy control group were significantly higher than in the non-treated diabetic group (26). In addition, both a cross-sectional report in Chile (29) and a population-based study (14) reported that AMH concentrations were lower in women with T1DM. However, few studies have focused on the AMH levels in patients with T2DM, which requires further research.

Therefore, our purpose of this study was to evaluate the overall effects of T2DM on ovarian reserve and pregnancy outcomes during the ART process, and provide improved guidance that would increase clinical ART pregnancy success rates.

## 2 Materials and methods

### 2.1 Study population

It is a retrospective cohort study that was performed at the Reproductive Medicine Special Hospital, The First Hospital of Lanzhou University, between January 2019 and December 2022. This study was reviewed and approved by the Ethics Committee of the First Hospital of Lanzhou University (LDYYLL2019-44). Based on the inclusion and exclusion criteria, a total of 265 infertile female patients were enrolled. The clinical and follow-up information of the first fresh embryo transfer cycle of the 265 patients was eventually analyzed.

### 2.2 Inclusion criteria and exclusion criteria

Infertile female patients aged 20–45 years who underwent *in vitro* fertilization-embryo transfer (IVF-ET), intracytoplasmic sperm injection-embryo transfer (ICSI-ET), or rescue intracytoplasmic sperm injection-embryo transfer (RICSI-ET) in the first fresh cycle for female factor or (and) male factor infertility during January 2019 and December 2022 were enrolled.

Subjects with any of the following were excluded: (1) impaired fasting glucose, impaired glucose tolerance, or a diagnosis of type 1 diabetes, gestational diabetes, or other specific types of diabetes or glycemic abnormality; (2) incomplete follow-up information; (3) chromosome abnormality or (and) genetic disorder; (4) infectious or immunological diseases or suspicion of malignancy; (5) history of ovarian surgery, polycystic ovarian syndrome (PCOS), sexual hormone application within 3 months, and other endocrine disorders such as abnormal thyroid function and so on;

All the subjects were divided into two groups: the T2DM population and the non-T2DM reference population. The diagnostic criteria for T2DM, as defined by the International Diabetes Federation, are a fasting plasma glucose (FPG) level ≥7.0 mmol/L, a glycosylated hemoglobin (HbA1c) ≥6.5%, or a previous diagnosis of T2DM, and then the reference population was randomly selected after frequency matching with the T2DM group in terms of age (4 controls per T2DM patient). The final study comprised 53 infertile women with T2DM and a reference population of 212 who had never been diagnosed with any type of diabetes or glycemic abnormality.

### 2.3 Determination of sample size

We used PASS (version 21.0.3) to estimate whether the sample size met the requirements for conducting this study. To achieve 90% power at a 0.05 significance level, it was calculated that the sample size required to complete the analysis was the largest when calculated for clinical pregnancy rates. Given the results of the pre-analysis, the clinical pregnancy rates in the diabetic and control groups were 0.3 and 0.55, respectively. Consequently, the minimal total sample size that can satisfy the analysis for the purpose of this study was estimated to be 51 in the T2DM group and 204 in the non-T2DM group, which means that the sample size we included was able to meet the needs of the present study.

### 2.4 Data sources and measurement

In both study groups, the clinical data included anthropometric parameters and laboratory assessments like hormone levels and metabolic parameters.

#### 2.4.1 Physical examination

Anthropometric parameters, including height, weight, and body mass index (BMI), were measured according to standardized protocols. BMI was defined as the weight in kilograms divided by the square of height in meters (kg/m^2^).

#### 2.4.2 Laboratory assessment

Day 2 of menstruation was defined as the basal day, and blood samples were collected on day 2 of the menstrual cycle from 8:00 to 10:00 a.m. Fasting serum was collected on the basal day for further detection of sexual hormonal levels such as serum estradiol (E2), progesterone (P), follicle stimulating hormone (FSH), and luteinizing hormone (LH), which were measured by chemiluminescence (Roche Diagnostics, Germany). Fasting blood glucose was measured by the hexokinase method (Beckman Coulter, USA). Biomarkers that primarily represent ovarian reserve function, including Anti-Müllerian Hormone (AMH) and antral follicle count (AFC), were also measured among all female participants. Serum AMH concentrations were measured with a fully automated AMH electrochemiluminescence assay (ECLIA; Elecsys AMH assay, Roche Diagnostics, Germany) on the Cobas e 801 analyzer. Antral follicles were defined as 2-10 mm follicles visible in the ovaries by a vaginal ultrasound machine at 2-4 d of the menstrual cycle, and the total number of follicles in the ovaries was calculated bilaterally. Insulin was measured using the Immulite immunoassay. We used the homeostasis Model Assessment of Insulin resistance (HOMA-IR index): HOMA-IR index = fasting blood glucose (FBG) (mmol/L) ×fasting serum insulin (FINS) (μU/ml)/22.5 (30). The cut-off point of HOMA-IR value for defining insulin resistance was suggested to be 2.69, owing to previous Chinese studies showed that the cut-off point of HOMA-IR value was more consistent with the diagnostic criteria of insulin resistance in Chinese patients with T2DM (31–33).

### 2.5 Study procedures

Ovarian stimulation protocols, oocyte retrieval, and fertilization were performed according to standardized protocols, as recommended by the American Society for Reproductive Medicine and Chinese guidelines. Ovarian stimulation was performed under a long GnRH agonist, GnRH antagonist, or PPOS regimen.

#### 2.5.1 Long GnRH agonist regimen

Human menopausal gonadotropin (HMG, Lizhu Group) and/or injectable urinary follicle stimulating hormone (Lishenbao, Lizhu Group) and/or recombinant human follicle stimulating hormone (Gonal-F, Merck Serono S.p.A.) were given intramuscularly for super-ovulation according to the basal condition and follicular development. Follicular development was monitored by dynamic ultrasound, and the levels of sex hormones FSH, LH, E2, and P were measured on the same day to assess the ovarian response to the ovulation stimulating drugs and to adjust the dosage of ovulation stimulating drugs. When at least 3 follicles reached 16mm in diameter or 2 follicles reached or exceeded 18mm in diameter, recombinant human chorionic gonadotropin (Azer, Merck Serono S.p.A.) 250 U with (or without) HCG (Zhuhai, Lizhu Group) 2000 U was given as a trigger.

#### 2.5.2 GnRH antagonist regimen

Recombinant follicle-stimulating hormone (FSH; Gonal-F, Merck Serono) was started on day 2 or 3 of the menstrual cycle based on basal blood FSH, LH, E2, and AFC with an initiating start-up dose of 112.5-225 U and adjusted according to ovarian response. A gonadotropin-releasing hormone antagonist (Cetrorelix 250 mg; Merck Serono) was started when the lead follicle exceeded 12 mm. Follicular maturation was induced by GnRH-α (Daffelin, France) 0.2 mg combined with urinary human chorionic gonadotropin(HCG)2000 IU when 2 or more follicles measured 18 mm or more.

#### 2.5.3 PPOS regimen

Oral dydrogesterone tablets (20 mg/d) were started on the 2nd to 5th menstrual day until the trigger day, and human menopausal gonadotrophin (HMG, Lizhu Group) and/or injectable urinary follicle stimulating hormone (Lishenbao, Lizhu Group) and/or recombinant human follicle stimulating hormone (Gonal-F, Merck Serono S.p.A.) were given intramuscularly for super-ovulation. The follicle size and the levels of sex hormones FSH, LH, E2, and P were monitored dynamically to assess ovarian responsiveness to ovulation stimulating drugs and to adjust the dosage of ovulation stimulating drugs. When at least 3 follicles reach 16mm in diameter or more than 2 follicles reach or exceed 18mm in diameter, human chorionic gonadotropin (HCG, Lizhu Group) 2000 U in combination with GnRH-a (Daffelin, France) 0.2mg was administered.

Oocyte retrieval was performed 34-36 hours later. Selection of different fertilization methods according to male semen parameters and infertility factors. Embryo transfer was performed 72 hours after oocyte retrieval.

### 2.6 Outcome measures and definition

The primary observation of ovarian reserve was AMH, and the main pregnancy outcome indicators included clinical pregnancy rate (CPR), live birth rate (LBR), and abortion rate (AR). MII oocyte rate, normal fertilization rate, and high-quality embryo rate were also compared to assess the effect of T2DM on oocyte maturation, fertilization, and embryo quality.

Clinical pregnancy rate (%) (CPR) = number of clinical pregnancy cycles/number of transplant cycles; A positive blood test for β-HCG 14 days after transplantation was considered a pregnancy; a clinical pregnancy was diagnosed on an ultrasound 30 days after transplantation if a gestational sac was observed. Live birth rate (%) (LBR) = number of live birth cycles/number of transplant cycles. Abortion rate (%) (AR) = number of abortion cycles/number of clinical pregnancy cycles; The fertilization rate for each individual patient was evaluated based on their insemination procedure. For patients who underwent conventional insemination IVF, the normal fertilization rate = 2PN/number of oocytes inseminated; For patients who underwent ICSI, the normal fertilization rate = 2PN/number of oocytes injected. High-quality embryo rate (%) = number of high-quality embryos per patient/number of available embryos per patient; MII oocyte rate (%) = number of MII oocytes per patient/number of oocytes retrieved per patient;

### 2.7 Statistical analysis

Statistical analysis was performed using SPSS 26.0; continuous variables were expressed as mean ± SD; quantitative variables were tested using the Mann–Whitney tests. Categorical variables were represented by frequency and percentage (%), and the differences between groups were assessed by the Pearson chi-square test (χ^2^-test) or Fisher exact probability method. Univariate and multivariate logistic regression were used to further investigate the effect of T2DM on clinical pregnancy outcomes. The binary outcome of this model was whether a clinical pregnancy, miscarriage, or live birth occurred. P < 0.05 was considered to indicate statistically significant differences.

## 3 Result

### 3.1 Baseline characteristics

We identified 53 infertile women with T2DM and selected 212 matched nondiabetic controls. The anthropometric and metabolic characteristics, sexual hormone, AMH levels and indicators related to diabetes and insulin resistance (IR) such as HbA1c, FINS, and HOMA-IR are shown in **Table 1**. The T2DM group had significantly higher BMIs and fasting blood glucose (26.4 ± 5.16 vs. 22.13 ± 2.67, P<0.001; 8.97 ± 3.78 vs. 4.60 ± 0.43, P<0.001, respectively) than the non-T2DM group. Furthermore, a significant decrease in AMH concentrations and basal progesterone levels was observed in patients with T2DM (2.43 ± 2.31 vs. 3.58 ± 2.58, P<0.001; 0.28 ± 0.27 vs. 0.53 ± 0.46, P<0.001, respectively; **Table 1**).

**Table 1:** Baseline characteristics of the study population.

Parameters	T2DM (n =53)	Non-T2DM (n=212)	P-value
Age (years)	32.87 ± 3.63	32.87 ± 3.61	1.000
BMI (kg/m^2^)	26.40 ± 5.16	22.13 ± 2.67	<0.001
Duration of infertility (years)	4.14 ± 2.56	4.13 ± 3.40	0.401
AMH (μ g/L)	2.43 ± 2.31	3.58 ± 2.58	<0.001
AFC (n)	11.24 ± 6.63	12.08 ± 5.73	0.244
Serum fasting glucose (mmol/L) HbA1c(%) FINS (mIU/L) HOMA-IR	8.97 ± 3.78 8.09 ± 2.13 13.10 ± 3.83 5.07 ± 3.28	4.60 ± 0.43 − − −	<0.001
Basal FSH (IU/L)	7.30 ± 3.40	7.29 ± 3.28	0.679
Basal LH (IU/L)	4.39 ± 2.37	4.62 ± 2.30	0.426
Basal E2 (ng/L)	35.59 ± 26.83	42.46 ± 32.32	0.112
Basal P (ng/mL)	0.28 ± 0.27	0.53 ± 0.46	<0.001

Values are expressed as mean ± standard deviation.

BMI, body mass index; AMH, anti-Müllerian hormone; AFC, antral follicle count; HbA1c, glycosylated hemoglobin; FINS, fasting serum insulin; HOMA-IR, homeostatic model assessment of insulin resistance; FSH, follicle stimulating hormone; LH, luteinizing hormone; E2, estradiol; P, progesterone; T2DM, type 2 diabetes mellitus.

HOMA-IR, Homeostatic model assessment of insulin resistance, cut-off >2.69 considered elevated.

### 3.2 Comparison of infertility and cycle stimulation characteristics between two groups

As presented in **Table 2**, regarding ovarian stimulation, there was a significant difference among the two groups in terms of total and initial doses of gonadotropin (GN); those with T2DM required significantly higher initial and total doses of gonadotropin to support follicular development but had fewer retrieved oocytes compared to the non-T2DM group. The duration of ovarian stimulation was higher in the T2DM group but without a significant between-group difference (12.15 ± 2.26 vs. 11.76 ± 2.44, P = 0.382). The etiological classification and methods of assisting pregnancy, as well as ovulation stimulation protocols, did not differ between the two groups.

**Table 2:** Infertility and cycle stimulation characteristics of the study population.

	T2DM (n =53)	Non-T2DM (n=212)	P-value
Type of infertility (%)			0.496
Primary infertility	32 (60.4)	117 (55.2)	
Secondary infertility	21 (39.6)	95 (44.8)	
Cause of infertility (%)			0.054
Male factors	2 (3.8)	35 (16.5)	
Female factors	41 (77.4)	132 (62.3)	
Multiple causes	10 (18.8)	42 (19.8)	
Unknown	0 (0)	3 (1.4)	
Method of ART (%)			0.097
IVF	40 (75.5)	126 (59.4)	
ICSI	10 (18.9)	65 (30.6)	
RICSI	3 (5.6)	21 (10)	
COH protocols (%)			0.081
Long agonist	31 (58.5)	138 (65.1)	
Short antagonist	7 (13.2)	28 (13.2)	
PPOS	15 (28.3)	34 (16.0)	
Others	0	12 (5.7)	
Days of stimulation (days)	12.15 ± 2.26	11.76 ± 2.44	0.382
Total GN dosage (IU)	3402 ± 1207	2788 ± 1195	<0.001
Initial GN dosage (IU)	251 ± 57	219 ± 56	<0.001
Oocytes retrieved (n)	10.58 ± 7.09	13.44 ± 6.52	0.005

Values are expressed as mean ± standard deviation or n (%).

ART, assisted reproductive technology; IVF, in vitro fertilization; ICSI, intracytoplasmic sperm injection; RICSI, rescue intracytoplasmic sperm injection; COH, controlled ovarian hyperstimulation; GN, gonadotrophin.

In order to further analyze the effect of blood glucose itself on the cycle stimulation characteristics such as stimulation time, GN doses and the number of oocytes retrieved during ovarian stimulation, we analyzed the correlation between blood glucose and the indicators of ovarian stimulation mentioned above. As shown in the **Supplementary Table 1**, blood glucose level was positively correlated with the total dose of gonadotrophin (GN), but no correlation was found between the blood glucose level and the number of oocytes retrieved or the time of ovarian stimulation.

### 3.3 The influence of T2DM on embryo development and pregnancy outcomes

The MII oocyte rate, fertilization rate, and high-quality embryo rate, as indicators of embryo development, were significantly lower in the T2DM group (86.8%, 56%, and 36.7%, respectively) compared with the non-T2DM group (89.9%, 63.4%, and 64.8%, respectively). The clinical pregnancy rate, abortion rate, and live birth rate, which represent pregnancy outcomes, are presented in **Table 3**. Apparently, by comparing the pregnancy outcomes of the two groups, patients in the T2DM group had significantly lower clinical pregnancy and live birth rates (30.2% vs. 52.8%, P = 0.003; 15.1% vs. 47.6%, P<0.001, respectively) but a significantly higher risk of miscarriage (50.0% vs. 9.8%, P<0.001) (**Table 3**).

**Table 3:** Embryonic development and pregnancy outcomes of the study population.

	T2DM (n =53)	Non-T2DM(n=212)	P-value
MII oocyte rate (%)	86.8 (487/561)	89.9 (2562/2849)	0.028
Fertilization rate (%)	56.0 (314/561)	63.4 (1807/2849)	0.001
High-quality embryo rate (%)	36.7 (73/199)	64.8 (860/1327)	<0.001
Clinical pregnancy rate (%)	30.2 (16/53)	52.8 (112/212)	0.003
Live birth rate (%)	15.1 (8/53)	47.6 (101/212)	<0.001
Abortion rate (%)	50.0 (8/16)	9.8 (11/112)	<0.001

Values are expressed as n (%).

T2DM, type 2 diabetes mellitus.

Since diabetes-related indicators such as HbA1c, FINS, and HOMA-IR were accessible within the T2DM group, we conducted a univariate logistic regression analysis to delve deeper into the influence of these indicators on assisted reproductive outcomes among women with T2DM (refer to **Supplementary Table 2**). The results of the logistic regression analysis indicated that there was no significant impact of diabetes-related indicators on assisted reproductive outcomes in women with T2DM.

### 3.4 T2DM is an independent risk factor affecting assisted pregnancy outcomes

To further investigate the effect of T2DM on clinical pregnancy outcomes, a binary logistic regression model was performed. Univariate logistic regression was performed to identify potential confounders affecting CPR, LBR, and AR (**Supplementary Tables 3**, **5**, **7**), and variables with a value of P<0.1 were included in the multivariable logistic regression analysis (**Supplementary Tables 4**, **6**, **8**). Due to the involvement of blood glucose levels in the diagnosis of diabetes, the collinearity between the two is strong, and the blood glucose level is strongly correlated with diabetes in clinical significance and statistics. Therefore, only the diabetes group is included in the multivariate regression analysis. The odds ratios (ORs) with 95% confidence intervals (CIs) and P-values of assisted pregnancy outcomes between two groups were calculated, the results are shown in **Table 4**.

**Table 4:** Logistic regression analysis on the effect of T2DM on pregnancy outcomes.

Pregnancy outcomes	Groups	Incidence rate	Unadjusted OR (95%CI)	P-value	Adjusted OR (95%CI)	P-value
CPR	Non-T2DM	52.8 (112/212)	Ref.	Ref.	Ref.	Ref.
	T2DM	30.2 (16/53)	0.386 (0.202-0.736)	0.004	0.458 (0.235-0.891)	0.022^a^
LBR	Non-T2DM	47.6 (101/212)	Ref.	Ref.	Ref.	Ref.
	T2DM	15.1 (8/53)	0.195 (0.088-0.434)	<0.001	0.227 (0.101-0.513)	<0.001^b^
AR	Non-T2DM	9.8 (11/112)	Ref.	Ref.	Ref.	Ref.
	T2DM	50.0 (8/16)	3.248 (1.236-8.538)	0.017	3.316 (1.248-8.811)	0.016^c^

Ref, reference; CPR, clinical pregnancy rate; LBR, live birth rate; AR, abortion rate; CI, confidence interval; OR, odds ratio; T2DM, type 2 diabetes mellitus.

a Adjusted by AMH, No. of oocytes retrieved, No. of MII oocytes, 2PN and transferrable embryos.

b Adjusted by BMI, AMH, infertility type, total dose of GN and time of ovarian stimulation, No. of oocytes retrieved, No. of MII oocytes, 2PN and transferrable embryos.

c Adjusted by age.

As shown in **Table 4**, T2DM is an independent risk factor for assisted reproductive outcomes, negatively affecting clinical pregnancy rate and live birth rate (adjusted odds ratio [a OR], 0.458, adjusted 95% confidence interval [CI], 0.235-0.891, P = 0.022; a OR, 0.227, 95% CI, 0.101-0.513, P<0.001);. The abortion rate in the T2DM group was 3.316 times higher than that in the non-T2DM group (a OR, 3.316, 95% CI, 1.248-8.811, P = 0.016; **Table 4**).

## 4 Discussion

In this four-year retrospective cohort study, we uniquely investigated clinical pregnancy rate (CPR), live birth rate (LBR), abortion rate (AR), and AMH concentrations among reproductive-aged women with T2DM who undergo ART treatments. The comparison of baseline parameters and assisted reproductive outcomes between the two groups showed that serum AMH levels decreased significantly and clinical pregnancy outcomes were poorer in the T2DM group, which demonstrated that T2DM adversely affects ovarian reserve and assisted reproductive outcomes.

The negative impact of diabetes on female fertility has been identified in several studies. A Chinese cohort study, comprising >2 million couples, reported that elevated pre-pregnancy maternal glucose levels were associated with the couple’s lower fecundability (34). Interestingly, another cohort study based on the Norwegian Mother and Child obtained the similar results (10). Besides, the incidence of spontaneous abortion (11) and the risk of pregnancy complications (35) are observed to be higher in diabetic women. The literature mentioned above strongly supports the results of this study, however, there are still some studies that remain controversial regarding the fertility in patients with T2DM, such as an observational, descriptive study in Iran that showed a higher rate of fertility in married women with T2DM (36). In addition to this, the study also found an unexpectedly low prevalence of polycystic ovary syndrome (PCOS) and hyperandrogenemia in the research group, which may be the reason why married women with T2DM have higher fertility rate. This is because PCOS was associated with increasing the probability of developing T2DM (9), and the onset of the two overlaps with each other. It is well known that PCOS is the most common endocrine disease in childbearing women, most often accompanied by metabolic and reproductive-related complications (37), such as anovulation and subfertility. Consequently, the reduced prevalence of PCOS in women with type 2 diabetes may lead to a pick-up in fertility.

Furthermore, regarding ovarian stimulation characteristics, we have found that the T2DM group required higher total and initial exogenous gonadotrophin (GN) dosage during the ART process. These findings may be the result of impaired ovarian sensitivity to exogenous GN stimulation under the influence of diabetes. A recent retrospective cohort study proposed that average GN dosage per follicle is a reliable index of ovarian response to exogenous GN, and lower exogenous GN requirements suggest the ovary response is more sensitive to GN stimulation (38). In addition, a longer duration of ovarian stimulation was also observed in the T2DM group despite lacks statistical significance. This may be due to the fact that the high total GN dosage, but not the duration of ovarian stimulation, is associated with decreasing the rate of live births in fresh cycles (39). Therefore, minimizing the total GN dose or limiting the duration of stimulation should be considered in fresh autologous cycles to improve pregnancy outcomes. Currently, there is a paucity of studies directly focused on the exogenous GN dosage and ovarian stimulation length in patients with T2DM, and thus further studies are necessary in the future.

Also, a significant decrease in the number of retrieved oocytes and poorer reproductive outcomes were observed in patients with T2DM; this might be influenced by the high glucose microenvironment during the progression of diabetes. Hyperglycemia induces the formation of reactive oxygen species (ROS) that promote oxidative stress and lead to mitochondrial dysfunction (7). Mitochondrion, as an energy source, provides energy for the development, maturation, and fertilization of oocytes. Meanwhile, the key step of steroid hormone biosynthesis also occurs in the mitochondria of granulosa cells, and accumulating evidence indicates that higher follicular estradiol levels correlate well with successful fertilization following ART (40). Moreover, the hyperglycemic condition may disturb the connexin expression and perturb the oocyte-granulosa gap junction communication (41) and through apoptosis, it can affect oocyte competence and pregnancy outcomes (42). Therefore, we speculate that hyperglycemia-induced mitochondrial dysfunction and granulosa cell apoptosis may contribute to the decline in quantity and quality of oocytes, a reduction in steroidogenesis, and a decreased fertilization rate, which ultimately jeopardize fertility.

AMH, produced by the granulosa cells of small ovarian follicles, represents the quantity and quality of follicles. Soto et al. (29) showed that AMH levels decreased earlier in diabetes mellitus (DM). Our data also showed a marked decline in women with T2DM. Several mechanisms may help explain the more rapid decline in ovarian reserve observed in diabetic women, including oxidative stress and NF-κB pathway activation, which have direct effects on granulosa cell injury (43). On the other hand, in the hyperglycemic state, a disturbed microenvironment may lead to the apoptosis of granulocytes. As one study has shown that elevated follicular glucose profoundly accelerated the pyroptosis of ovarian granulosa cells, affecting the synthesis of hormones (44) and AMH secretion. Thus, this may be another important reason for the decline in AMH levels in diabetic patients. Of course, due to the limitations of human knowledge, there may be many other unknown mechanisms that lead to diabetes-related reproductive dysfunction and decreased ovarian reserve, which need to be further explored in future studies.

As this was a retrospective study, a major limitation was the possibility of the potential bias due to missing data or incomplete medical records. Furthermore, another limitation of this study was a lack of detailed population characteristics, such as smoking and drinking status, as well as the duration of diabetes and the presence of diabetes-related complications, these indicators may affect fertility outcomes as unknown confounding factors. Finally, as in any cohort study, selection bias cannot be excluded. To minimize the limitations and get a more creditable conclusion, we consider conducting multi-center, prospective studies with larger sample sizes and more detailed medical data records in the future.

On the other hand, this study had certain strengths. All study populations were matched by age, as it is well known that age is the most critical factor affecting AMH levels. In addition to this, age was also adjusted in multivariate analysis, which achieved a doubly robust test or estimation. Lastly, our study uniquely investigated ovarian reserve and assisted reproductive outcomes in infertile women with T2DM requiring ART treatments during the reproductive years, the results may help to improve the success rate of assisted reproductive therapy.

In summary, our study concludes the adverse effects of T2DM on ovarian reserve and assisted reproductive outcomes during the ART process, emphasizes the importance of pre-pregnancy blood glucose screening and control for pre-pregnancy health care, and highlights that early intervention and preventive treatment for female partners with T2DM before ART treatment are necessary. Meanwhile, it is also a key measure to improve assisted reproductive outcomes and promote maternal and newborn health in the future.

## Data availability statement

The original contributions presented in the study are included in the article/**Supplementary Material**. Further inquiries can be directed to the corresponding author.

## Ethics statement

This study was reviewed and approved by the Ethics Committee of the First Hospital of Lanzhou University (LDYYLL2019-44). The studies were conducted in accordance with the local legislation and institutional requirements. The participants provided their written informed consent to participate in this study.

## Author contributions

XQ and XL contributed to the central idea and design of the work. YL, QZ, and YXL guided the writing of the article. XQ, JD, and RH assisted in the analysis and interpretation of data. HL and XL provided critical feedback on the manuscript. The original draft was written by XQ. All authors contributed to the article and approved the submitted version.

## Supplementary material

Supplementary Table 1 Correlation between blood glucose levels with ovarian stimulation characteristics.

Supplementary Table 2 Univariate logistic regression analysis of diabetes-related indicators and assisted reproductive outcomes in the T2DM group.

Supplementary Table 3 Univariate logistic regression analysis on the clinical pregnancy rate (CPR).

Supplementary Table 4 Multivariate analysis on the clinical pregnancy rate (CPR) with backward approach regression.

Supplementary Table 5 Univariate logistic regression analysis on the live birth rate (LBR).

Supplementary Table 6 Multivariate analysis on the live birth rate (LBR) with backward approach regression.

Supplementary Table 7 Univariate logistic regression analysis on the abortion rate (AR).

Supplementary Table 8 Multivariate analysis on the abortion rate (AR) with backward approach regression.

## References

[1] Nagore E Martinez-Garcia MA Gomez-Olivas JD Manrique-Silva E Martorell A Bañuls J. Relationship between type 2 diabetes mellitus and markers of cutaneous melanoma aggressiveness: an observational multicentric study in 443 patients with melanoma*. Br J Dermatol (2021) 185(4):756–63. doi: 10.1111/bjd.19813 33453061

[2] Wang X Xian T Jia X Zhang L Liu L Man F. A cross-sectional study on the associations of insulin resistance with sex hormone, abnormal lipid metabolism in T2dm and igt patients. Medicine (2017) 96(26):e7378. doi: 10.1097/MD.0000000000007378 28658166 PMC5500088

[3] Zhou T Hu Z Yang S Sun L Yu Z Wang G. Role of adaptive and innate immunity in type 2 diabetes mellitus. J Diabetes Res (2018) 2018:1–9. doi: 10.1155/2018/7457269 PMC6250017 30533447

[4] Chatterjee S Khunti K Davies MJ. Type 2 diabetes. Lancet (2017) 389(10085):2239–51. doi: 10.1016/S0140-6736(17)30058-2 28190580

[5] Wang W-Y Liu X Gao X-Q Li X Fang Z-Z. Relationship between acylcarnitine and the risk of retinopathy in type 2 diabetes mellitus. Front Endocrinol (2022) 13:834205. doi: 10.3389/fendo.2022.834205 PMC8964487 35370967

[6] Creţu D Cernea S Onea CR Pop R-M. Reproductive health in women with type 2 diabetes mellitus. Hormones (2020) 19(3):291–300. doi: 10.1007/s42000-020-00225-7 32613536

[7] Daryabor G Atashzar MR Kabelitz D Meri S Kalantar K. The effects of type 2 diabetes mellitus on organ metabolism and the immune system. Front Immunol (2020) 11:1582. doi: 10.3389/fimmu.2020.01582 32793223 PMC7387426

[8] Yu Y Li J Jiang Y Zaid M Zhao Q Wang N. Association between reproductive factors and type 2 diabetes: A cross-sectional study. Int J Environ Res Public Health (2022) 19(2):1019. doi: 10.3390/ijerph19021019 35055839 PMC8775663

[9] Thong EP Codner E Laven JSE Teede H. Diabetes: A metabolic and reproductive disorder in women. Lancet Diabetes Endocrinol (2020) 8(2):134–49. doi: 10.1016/S2213-8587(19)30345-6 31635966

[10] Whitworth KW Baird DD Stene LC Skjaerven R Longnecker MP. Fecundability among women with type 1 and type 2 diabetes in the norwegian mother and child cohort study. Diabetologia (2011) 54(3):516–22. doi: 10.1007/s00125-010-2003-6 PMC3650679 21170514

[11] McGrogan A Snowball J de Vries CS. Pregnancy losses in women with type 1 or type 2 diabetes in the uk: an investigation using primary care records. Diabetes Med (2014) 31(3):357–65. doi: 10.1111/dme.12332 24111989

[12] Diagnosis and classification of diabetes mellitus. Diabetes Care (2013) 36 Suppl 1(Suppl 1):S67–74. doi: 10.2337/dc13-S067 PMC3537273 23264425

[13] Codner E Merino PM Tena-Sempere M. Female reproduction and type 1 diabetes: from mechanisms to clinical findings. Hum Reprod Update (2012) 18(5):568–85. doi: 10.1093/humupd/dms024 22709979

[14] Kim C Karvonen-Gutierrez C Kong S Arends V Steffes M McConnell DS. Antimüllerian Hormone among Women with and without Type 1 Diabetes: The Epidemiology of Diabetes Interventions and Complications Study and the Michigan Bone Health and Metabolism Study. Fertil Steril (2016) 106(6):1446–52. doi: 10.1016/j.fertnstert.2016.07.009 PMC5159208 27475411

[15] Codner E Escobar-Morreale HF. Clinical review: hyperandrogenism and polycystic ovary syndrome in women with type 1 diabetes mellitus. J Clin Endocrinol Metab (2007) 92(4):1209–16. doi: 10.1210/jc.2006-2641 17284617

[16] Strotmeyer ES Steenkiste AR Foley TP Jr. Berga SL Dorman JS. Menstrual cycle differences between women with type 1 diabetes and women without diabetes. Diabetes Care (2003) 26(4):1016–21. doi: 10.2337/diacare.26.4.1016 12663566

[17] Gaete X Vivanco M Eyzaguirre FC López P Rhumie HK Unanue N. Menstrual cycle irregularities and their relationship with hba1c and insulin dose in adolescents with type 1 diabetes mellitus. Fertil Steril (2010) 94(5):1822–6. doi: 10.1016/j.fertnstert.2009.08.039 19796762

[18] Sjöberg L Pitkäniemi J Haapala L Kaaja R Tuomilehto J. Fertility in people with childhood-onset type 1 diabetes. Diabetologia (2013) 56(1):78–81. doi: 10.1007/s00125-012-2731-x 23011355

[19] Jonasson JM Brismar K Sparén P Lambe M Nyrén O Ostenson CG. Fertility in women with type 1 diabetes: A population-based cohort study in Sweden. Diabetes Care (2007) 30(9):2271–6. doi: 10.2337/dc06-2574 17563340

[20] Lorenzen T Pociot F Johannesen J Kristiansen OP Nerup J. A population-based survey of frequencies of self-reported spontaneous and induced abortions in danish women with type 1 diabetes mellitus. Danish iddm epidemiology and genetics group. Diabetes Med (1999) 16(6):472–6. doi: 10.1046/j.1464-5491.1999.00087.x 10391394

[21] Artasensi A Pedretti A Vistoli G Fumagalli L. Type 2 diabetes mellitus: A review of multi-target drugs. Molecules (2020) 25(8):1987. doi: 10.3390/molecules25081987 32340373 PMC7221535

[22] Lambrinoudaki I Paschou SA Armeni E Goulis DG. The interplay between diabetes mellitus and menopause: clinical implications. Nat Rev Endocrinol (2022) 18(10):608–22. doi: 10.1038/s41574-022-00708-0 35798847

[23] Mattsson K Nilsson-Condori E Elmerstig E Vassard D Schmidt L Ziebe S. Fertility outcomes in women with pre-existing type 2 diabetes—a prospective cohort study. Fertility Sterility (2021) 116(2):505–13. doi: 10.1016/j.fertnstert.2021.02.009 34353572

[24] Qi L Liu Y-p Wang S-m Shi H Chen X-l Wang N-n. Abnormal bmi in male and/or female partners are deleterious for embryonic development and pregnancy outcome during art process: A retrospective study. Front Endocrinol (2022) 13:856667. doi: 10.3389/fendo.2022.856667 PMC9068983 35528007

[25] Brouwer J Laven JSE Hazes JMW Schipper I Dolhain RJEM. Levels of serum anti-müllerian hormone, a marker for ovarian reserve, in women with rheumatoid arthritis: serum amh and ovarian reserve in women with ra. Arthritis Care Res (2013) 65(9):1534–8. doi: 10.1002/acr.22013 23554429

[26] Nayki U Onk D Balci G Nayki C Onk A Gunay M. The effects of diabetes mellitus on ovarian injury and reserve: an experimental study. Gynecologic Obstetric Invest (2016) 81(5):424–9. doi: 10.1159/000442287 26682912

[27] Cedars MI. Evaluation of female fertility—Amh and ovarian reserve testing. J Clin Endocrinol Metab (2022) 107(6):1510–9. doi: 10.1210/clinem/dgac039 35100616

[28] Yang W Lin C Zhang M Lv F Zhu X Han X. Assessment of ovarian reserve in patients with type 1 diabetes: A systematic review and meta-analysis. Endocrine (2022) 77(2):205–12. doi: 10.1007/s12020-022-03091-y 35637405

[29] Soto N Iñiguez G López P Larenas G Mujica V Rey RA. Anti-müllerian hormone and inhibin B levels as markers of premature ovarian aging and transition to menopause in type 1 diabetes mellitus. Hum Reprod (2009) 24(11):2838–44. doi: 10.1093/humrep/dep276 19643804

[30] Yang X Hu R Wang Z Hou Y Song G. Associations between serum folate level and homa-ir in chinese patients with type 2 diabetes mellitus. Diabetes Metab Syndr Obes (2023) 16:1481–91. doi: 10.2147/dmso.S409291 PMC10204713 37229352

[31] Zhou M Zhu L Cui X Feng L Zhao X He S. The triglyceride to high-density lipoprotein cholesterol (Tg/hdl-C) ratio as a predictor of insulin resistance but not of β Cell function in a chinese population with different glucose tolerance status. Lipids Health Dis (2016) 15:104. doi: 10.1186/s12944-016-0270-z 27267043 PMC4895977

[32] Yang WB Wang HL Mao JT Chen Z Xu JW Wang LH. The correlation between ct features and insulin resistance levels in patients with T2dm complicated with primary pulmonary tuberculosis. J Cell Physiol (2020) 235(12):9370–7. doi: 10.1002/jcp.29741 32346889

[33] Wang Z Wang YJ Liu ZY Li Q Kong YW Chen YW. Effect of insulin resistance on recurrence after radiofrequency catheter ablation in patients with atrial fibrillation. Cardiovasc Drugs Ther (2023) 37(4):705–13. doi: 10.1007/s10557-022-07317-z 35218469

[34] Zhao J Hong X Zhang H Dai Q Huang K Zhang X. Pre-pregnancy maternal fasting plasma glucose levels in relation to time to pregnancy among the couples attempting first pregnancy. Hum Reprod (2019) 34(7):1325–33. doi: 10.1093/humrep/dez069 PMC6613343 31216361

[35] Ringholm L Damm P Mathiesen ER. Improving pregnancy outcomes in women with diabetes mellitus: modern management. Nat Rev Endocrinol (2019) 15(7):406–16. doi: 10.1038/s41574-019-0197-3 30948803

[36] Tavakolian Arjmand A Nouri M Tavakolian Arjmand S. Surprisingly low infertility rate in married type 2 diabetic women: A rather curious paradox to the current opinion of insulin resistance as the joint pathogenesis of poly cystic ovary syndrome and type 2 diabetes mellitus. Diabetes Metab Syndr (2015) 9(4):201–4. doi: 10.1016/j.dsx.2015.08.007 26364227

[37] Łebkowska A Adamska A Karczewska-Kupczewska M Nikołajuk A Otziomek E Milewski R. Serum anti-müllerian hormone concentration in women with polycystic ovary syndrome and type 1 diabetes mellitus. Metabolism (2016) 65(5):804–11. doi: 10.1016/j.metabol.2016.02.005 26961579

[38] Liu S Ma S Li Y. The average gonadotrophin dosage per follicle is predictive of ovarian response and cumulative live birth chances after in vitro fertilization: A retrospective cohort study. BMC Womens Health (2023) 23(1):45. doi: 10.1186/s12905-023-02195-5 36739381 PMC9898889

[39] Gerber RS Fazzari M Kappy M Cohen A Galperin S Lieman H. Differential impact of controlled ovarian hyperstimulation on live birth rate in fresh versus frozen embryo transfer cycles: A society for assisted reproductive technology clinic outcome system study. Fertil Steril (2020) 114(6):1225–31. doi: 10.1016/j.fertnstert.2020.06.021 33012553

[40] Sreerangaraja Urs DB Wu WH Komrskova K Postlerova P Lin YF Tzeng CR. Mitochondrial function in modulating human granulosa cell steroidogenesis and female fertility. Int J Mol Sci (2020) 21(10):3592. doi: 10.3390/ijms21103592 32438750 PMC7279321

[41] Ratchford AM Esguerra CR Moley KH. Decreased oocyte-granulosa cell gap junction communication and connexin expression in a type 1 diabetic mouse model. Mol Endocrinol (2008) 22(12):2643–54. doi: 10.1210/me.2007-0495 PMC2626198 18829945

[42] Predheepan D Daddangadi A Uppangala S Laxminarayana SLK Raval K Kalthur G. Experimentally induced hyperglycemia in prepubertal phase impairs oocyte quality and functionality in adult mice. Endocrinology (2022) 163(9):bqac121. doi: 10.1210/endocr/bqac121 35917567

[43] Pala HG Pala EE Artunc Ulkumen B Aktug H Yavasoglu A Korkmaz HA. The protective effect of granulocyte colony-stimulating factor on endometrium and ovary in a rat model of diabetes mellitus. Gynecol Obstet Invest (2014) 78(2):94–100. doi: 10.1159/000363239 25033771

[44] Xu R Zhao H Qi J Yao G He Y Lu Y. Local glucose elevation activates pyroptosis *via* nlrp3 inflammasome in ovarian granulosa cells of overweight patients. FASEB J (2023) 37(3):e22807. doi: 10.1096/fj.202201796RR 36826432

