# Supplementary material for: Adverse effects of type 2 diabetes mellitus on ovarian reserve and pregnancy outcomes during the assisted reproductive technology process
Source: Front Endocrinol (Lausanne). 2023 Nov 15;14:1274327. doi: 10.3389/fendo.2023.1274327 (PMC10686411; doi:10.3389/fendo.2023.1274327)
Supplement: Supplementary Table 1 — Correlation between blood glucose levels with ovarian stimulation characteristics. [file Table_1.docx]

**Supplemental tables**

**Supplemental table 1**

Correlation between blood glucose levels with ovarian stimulation characteristics

|  | r | P-value |
| --- | --- | --- |
| Retrieved oocytes number | -0.085 | 0.169 |
| Ovarian stimulation time | 0.092 | 0.137 |
| Total GN dosage | 0.184 | 0.003 |
| Initial GN dosage | 0.101 | 0.102 |

**Abbreviations:** GN, gonadotrophin; r, correlation coefficient.

**Supplemental table 2**

Univariate logistic regression analysis of diabetes-related indicators and assisted reproductive outcomes in the T2DM group

| Diabetes-related indicators | CPR  OR (95%CI) | P-value | LBR  OR (95%CI) | P-value | AR  OR (95%CI) | P-value |
| --- | --- | --- | --- | --- | --- | --- |
| HbA1c  FINS  HOMA-IR | 0.919 (0.689-1.244)  0.962 (0.820-1.129)  1.076 (0.904-1.282) | 0.562  0.638  0.408 | 0.846 (0.566-1.264)  0.911 (0.728-1.139)  0.966 (0.758-1.232) | 0.415  0.412  0.782 | 1.015 (0.713-1.444)  1.022 (0.842-1.240)  1.148 (0.933-1.143) | 0.936  0.827  0.193 |

**Abbreviations:** CPR, clinical pregnancy rate; LBR, live birth rate; AR, abortion rate; CI, confidence interval; OR, odds ratio; HbA1c, glycosylated hemoglobin; FINS, fasting serum insulin; HOMA-IR, homeostatic model assessment of insulin resistance; T2DM, type 2 diabetes mellitus.

**Supplemental table 3**

Univariate logistic regression analysis on the clinical pregnancy rate (CPR).

| Items | OR (95%CI) | P-value |
| --- | --- | --- |
| T2DM groups | 0.386 (0.202-0.736) | 0.004 |
| Age | 1.010 (0.945-1.080) | 0.761 |
| AMH | 1.102 (0.999-1.216) | 0.053 |
| AFC | 1.013 (0.972-1.056) | 0.533 |
| Basal FSH | 1.036 (0.956-1.123) | 0.383 |
| Basal LH | 1.015 (0.911-1.130) | 0.790 |
| Basal E2 | 0.993 (0.984-1.001) | 0.105 |
| Basal P | 1.392 (0.657-2.946) | 0.388 |
| FBG | 0.958 (0.866-1.059) | 0.401 |
| Duration of infertility | 1.025 (0.951-1.105) | 0.513 |
| Type of infertility | 1.275 (0.783-2.075) | 0.329 |
| Cause of infertility | 0.909 (0.682-1.212) | 0.515 |
| Method of ART | 0.927 (0.706-1.217) | 0.586 |
| COH protocols | 0.886 (0.686-1.146) | 0.358 |
| Days of stimulation | 0.956 (0.864-1.057) | 0.378 |
| Total GN dosage | 0.988 (0.882-1.014) | 0.308 |
| Initial GN dosage | 0.998 (0.994-1.003) | 0.455 |
| Number of Oocytes retrieved | 1.039 (1.001-1.078) | 0.042 |
| Number of MII oocytes | 1.041 (1.003-1.081) | 0.035 |
| Number of 2PN | 1.066 (1.016-1.118) | 0.009 |
| Number of available embryos | 1.098 (1.027-1.173) | 0.006 |

**Abbreviations:** AMH, anti-Müllerian hormone; AFC, antral follicle count; FSH, follicle stimulating hormone; LH, luteinizing hormone; E2, estradiol; P, progesterone; FBG, fasting blood glucose; ART, assisted reproductive technology; COH, controlled ovarian hyperstimulation; GN, gonadotrophin; T2DM, type 2 diabetes mellitus; CI, confidence interval; OR, odds ratio.

**Supplemental table 4**

Multivariate analysis on the clinical pregnancy rate (CPR) with backward approach regression.

| Variables |  | P-value | OR | 95% CI |
| --- | --- | --- | --- | --- |
| Variables in equation | T2DM groups | 0.022 | 0.458 | 0.235-0.891 |
|  | Number of available embryos | 0.034 | 1.077 | 1.006-1.153 |
| Variables not in equation | AMH | 0.505 |  |  |
|  | Number of oocytes retrieved | 0.871 |  |  |
|  | Number of MII oocytes | 0.834 |  |  |
|  | Number of 2PN | 0.370 |  |  |

**Abbreviations:** T2DM, type 2 diabetes mellitus; AMH, anti-Müllerian hormone; CI, confidence interval; OR, odds ratio;

**Supplemental table 5**

Univariate logistic regression analysis on the live birth rate (LBR).

| Items | OR (95%CI) | P-value |
| --- | --- | --- |
| T2DM groups | 0.195 (0.088-0.434) | <0.001 |
| Age | 1.045 (0.976-1.120) | 0.208 |
| BMI | 0.902 (0.832-0.977) | 0.011 |
| AMH | 1.106 (1.003-1.218) | 0.043 |
| AFC | 1.004 (0.963-1.047) | 0.848 |
| Basal FSH | 1.044 (0.964-1.132) | 0.288 |
| Basal LH | 0.983 (0.882-1.096) | 0.759 |
| Basal E2 | 0.995 (0.987-1.004) | 0.296 |
| Basal P | 1.842 (0.855-3.969) | 0.119 |
| FBG | 0.813 (0.699-0.945) | 0.007 |
| Duration of infertility | 1.014 (0.941-1.093) | 0.718 |
| Type of infertility | 1.571 (0.958-2.577) | 0.074 |
| Cause of infertility | 1.029 (0.770-1.376) | 0.847 |
| Method of ART | 0.960 (0.731-1.261) | 0.770 |
| COH protocols | 0.936 (0.721-1.215) | 0.621 |
| Days of stimulation | 0.915 (0.824-1.016) | 0.095 |
| Total GN dosage | 0.967 (0.816-1.013) | 0.058 |
| Initial GN dosage | 0.998 (0.994-1.002) | 0.320 |
| Number of Oocytes retrieved | 1.034 (0.997-1.073) | 0.071 |
| Number of MII oocytes | 1.034 (0.964-1.047) | 0.080 |
| Number of 2PN | 1.061 (1.012-1.113) | 0.015 |
| Number of available embryos | 1.102 (1.031-1.177) | 0.004 |

**Abbreviations:** T2DM, type 2 diabetes mellitus; BMI, body mass index; AMH, anti-Müllerian hormone; AFC, antral follicle count; FSH, follicle stimulating hormone; LH, luteinizing hormone; E2, estradiol; P, progesterone; FBG, fasting blood glucose; ART, assisted reproductive technology; COH, controlled ovarian hyperstimulation; GN, gonadotrophin; CI, confidence interval; OR, odds ratio.

**Supplemental table 6**

Multivariate analysis on the live birth rate (LBR) with backward approach regression.

| Variables |  | P-value | OR | 95% CI |
| --- | --- | --- | --- | --- |
| Variables in equation | T2DM groups | <0.001 | 0.227 | 0.101-0.513 |
|  | Number of available embryos | 0.057 | 1.069 | 0.998-1.145 |
| Variables not in equation | BMI | 0.453 |  |  |
|  | AMH | 0.544 |  |  |
|  | Type of infertility | 0.111 |  |  |
|  | Days of stimulation | 0.136 |  |  |
|  | Total GN dosage | 0.454 |  |  |
|  | Number of Oocytes retrieved | 0.797 |  |  |
|  | Number of MII oocytes | 0.676 |  |  |
|  | Number of 2PN | 0.588 |  |  |

**Abbreviations:** T2DM, type 2 diabetes mellitus; BMI, body mass index; AMH, anti-Müllerian hormone; GN, gonadotrophin; CI, confidence interval; OR, odds ratio.

**Supplemental table 7**

Univariate logistic regression analysis on the abortion rate (AR).

| Items | OR (95%CI) | P-value |
| --- | --- | --- |
| T2DM groups | 3.248 (1.236-8.538) | 0.017 |
| Age | 0.889 (0.782-1.001) | 0.072 |
| BMI | 0.988 (0.866-1.127) | 0.859 |
| AMH | 0.989 (0.821-1.191) | 0.904 |
| AFC | 1.034 (0.957-1.118) | 0.396 |
| Basal FSH | 0.976 (0.835-1.140) | 0.756 |
| Basal LH | 1.110 (0.917-1.344) | 0.284 |
| Basal E2 | 0.988 (0.967-1.009) | 0.252 |
| Basal P | 0.039 (0.001-2.009) | 0.107 |
| FBG | 1.245 (1.102-1.406) | <0.001 |
| Duration of infertility | 1.039 (0.912-1.184) | 0.562 |
| Type of infertility | 0.449 (0.157-1.285) | 0.135 |
| Cause of infertility | 0.545 (0.255-1.165) | 0.117 |
| Method of ART | 0.834 (0.420-1.657) | 0.604 |
| COH protocols | 0.789 (0.451-1.378) | 0.404 |
| Days of stimulation | 1.160 (0.955-1.409) | 0.134 |
| Total GN dosage | 0.998 (0.998-1.001) | 0.100 |
| Initial GN dosage | 0.002 (0.994-1.010) | 0.652 |
| Number of Oocytes retrieved | 1.018 (0.951-1.090) | 0.607 |
| Number of MII oocytes | 1.027 (0.959-1.101) | 0.446 |
| Number of 2PN | 1.018 (0.932-1.111) | 0.697 |
| Number of available embryos | 0.991 (0.876-1.121) | 0.881 |

**Abbreviations:** T2DM, type 2 diabetes mellitus; BMI, body mass index; AMH, anti-Müllerian hormone; AFC, antral follicle count; FSH, follicle stimulating hormone; LH, luteinizing hormone; E2, estradiol; P, progesterone; FBG, fasting blood glucose; ART, assisted reproductive technology; COH, controlled ovarian hyperstimulation; GN, gonadotrophin; CI, confidence interval; OR, odds ratio;

**Supplemental table 8**

Multivariate analysis on the abortion rate (AR) with backward approach regression.

| Variables |  | P-value | OR | 95% CI |
| --- | --- | --- | --- | --- |
| Variables in equation | T2DM groups | 0.016 | 3.316 | 1.248-8.811 |
| Variables not in equation | Age | 0.069 | 0.886 | 0.778-1.009 |

**Abbreviations:** T2DM, type 2 diabetes mellitus; CI, confidence interval; OR, odds ratio.
